# Untangling the corruption maze: exploring the complexity of corruption in the health sector

**DOI:** 10.1186/s13561-024-00530-6

**Published:** 2024-07-12

**Authors:** Margit Sommersguter-Reichmann, Gerhard Reichmann

**Affiliations:** 1 Department of Finance, University of Graz, Graz, Austria; 2 Department of Operations and Information Systems, University of Graz, Graz, Austria

**Keywords:** Corruption, Review, Typology, Institutional Corruption, Health, Manifestations

## Abstract

**Background:**

Healthcare corruption poses a significant threat to individuals, institutions, sectors, and states. Combating corruption is paramount for protecting patients, maintaining the healthcare system's integrity, and preserving public trust. As corruption evolves, takes new forms, and adapts to changing socio-political landscapes, understanding its manifestations is critical to developing effective anti-corruption strategies at individual and institutional levels.

**Objective:**

The aim was to comprehensively collate the manifestations of different types of corruption in healthcare to illustrate prevailing patterns and trends and to provide policymakers, practitioners, and researchers with practical insights to inform research agendas, regulatory and governance strategies, and accountability measures.

**Method:**

We conducted a narrative review of scientific articles published between 2013 and 2022 using keyword searches in SCOPUS and EBSCO. We utilized the corruption typology proposed by the European Union and Thompson's Institutional Corruption Framework to systematically identify manifestations across different corruption types. The Prisma scheme was employed to document the selection process and ensure reproducibility.

**Findings:**

Bribery in medical service provision was the most frequently investigated form of corruption, revealing rather uniform manifestations. Misuse of high-level positions and networks and institutional corruption also received considerable attention, with a wide range of misconduct identified in institutional corruption. Extending the analysis to institutional corruption also deepened the understanding of misconduct in the context of improper marketing relations and highlighted the involvement of various stakeholders, including academia. The pandemic exacerbated the vulnerability of the healthcare sector to procurement corruption. Also, it fostered new types of misconduct related to the misuse of high-level positions and networks and fraud and embezzlement of medical drugs, devices, and services.

**Conclusions:**

The review spotlights criminal actions by individuals and networks and marks a notable shift towards systemic misconduct within specific types of corruption. The findings highlight the necessity of customized anti-corruption strategies throughout the healthcare sector. These insights are crucial for policymakers, practitioners, and researchers in guiding the formulation of legal frameworks at local and global levels, governance strategies, and research priorities.

**Supplementary Information:**

The online version contains supplementary material available at 10.1186/s13561-024-00530-6.

## Introduction

Corruption in its various forms is a persistent problem across countries and industries, including health care. Corruption, however, is contextually manifest. Unique corruption cases in the health sector emerge due to particular characteristics of nations, regional settings, health systems, and the nature of the stakeholders involved. Savedoff, Hussmann [30] highlight uncertainty, asymmetric information, and numerous interacting stakeholders with diverse and divergent interests as specific characteristics of the health sector that form a breeding ground for corruption.

Scholars [33, 40] and organizations [4, 5, 7] have thus put forward diverse classifications to capture the complexity and nuances of corruption in health care to develop effective remedies. Underlying these efforts is the idea that effective countermeasures require a comprehensive understanding of the complexity of corruption, its influencing factors, and nested effects.

Transparency International [38], an umbrella organization with over 100 national chapters that combat corruption in their home countries, defines corruption as 'the abuse of entrusted power for private gain' [39]. In 2013, the European Union (EU) released a comprehensive corruption typology covering six categories of (primarily criminal) misconduct tailored to the healthcare sector and its stakeholders [4]. Conflicts of interest (COI), i.e. circumstances that risk a secondary interest influencing a primary interest unduly [34], represent drivers in each category. Another strand of corruption research that goes beyond the issues of criminal conduct and COI gained momentum with Thompson's seminal work on institutional corruption (InstCorr), which he derived from the nature of corruption in the US Congress [35]. InstCorr results from behavior that is a necessary or desirable part of institutional duties that nonetheless undermines its overall purpose. Thompson [36] contrasts InstCorr with individual corruption, noting that the latter predominantly represents criminal conduct. Lessig [18] vividly characterizes InstCorr, likening it to a magnet that causes a compass needle to no longer indicate the magnetic north. So far, extensive research on InstCorr has been conducted mainly in the pharmaceutical industry, including the undue influence of pharma on treatment decisions [26].

The scholarly literature highlights that corruption significantly affects health outcomes such as mortality (or life expectancy) and health status. For instance, Hanf et al. [10] explored the relationship between under-five mortality rates and Transparency International's Corruption Perception Index across 178 countries. They estimated that corruption indirectly contributes to 140,000 child deaths annually. Similarly, a Turkish study aggregating actual corruption incidences into a country-specific corruption index finds that corruption increases infant mortality rates in the long run [3]. Other studies link perceived and experienced corruption [43, 31] with poorer mental health outcomes. Still, others highlight the exacerbation of disaster-related deaths due to corruption in events like droughts, floods, and earthquakes [2]. SARS-CoV-2 drew attention to the negative impact of corruption on incidence, mortality [15], and vaccination rates [8] during a pandemic, a problem that low-income countries have been struggling with for much longer [25, 32].

Grasping the extent of corruption in general or in the health sector, in particular, using cost estimates is challenging in the case of individual corruption because of its hidden nature and InstCorr because of the complex acts with their multiple intertwined effects. The European Commission outlined that corruption costs the European Union (EU) member states between € 179 billion and € 990 billion annually, corresponding to 1.08 to 5.9 percent of its gross domestic product (GDP) [6]. Another study estimating the cost of healthcare fraud reports an average loss of 6.2% of global healthcare spending (€ 5.65 trillion), thus amounting to € 350 billion in absolute terms [9]. As the indirect economic, social, and political consequences of individual corruption are challenging to measure and InstCorr has rarely been addressed, the total costs of corruption are likely much higher than its estimates. However, apart from economic losses, corrupt actions impair the public's trust in the state, social cohesion, willingness to abide by the rule of law, and social development.

These negative consequences underscore the urgent need for comprehensive efforts to prevent and combat corruption at all levels. Several countries, such as the United States (US) with the Foreign Corrupt Practices Act and the 2010 Physician Payments Sunshine Act, the United Kingdom (UK) with the 2010 Bribery Act, and Germany with its 2015 Healthcare Corruption Prevention Act, to mention a few, have implemented solid general and health sector-specific anti-corruption laws. For low- and middle-income countries, these laws can serve as models for pushing the enactment of pertinent laws in these countries.

The scholarly literature offers different perspectives on the phenomenon, contributing to a comprehensive understanding of its complexity and involved stakeholders, including providers, patients, private firms, regulatory bodies, and research [12–14, 17, 19, 21, 29, 40] and facilitating the development of effective remedies [41, 16, 22, 27]. As corruption can involve individual and institutional wrongdoing, implementing context-specific anti-corruption legislation and other regulations and, when necessary, revising institutional frameworks to combat corruption effectively are required. The healthcare sector, however, has given little attention to addressing InstCorr, likely because of the challenges of identifying and mitigating it.

The importance of addressing corruption in the health sector, with its far-reaching negative impact on individuals, communities, and societies, cannot be overstated. As corruption evolves, takes new forms, and adapts to changing socio-political landscapes, understanding its manifestations is critical to developing effective anti-corruption strategies at individual and institutional levels. The need to oversee corrupt activities to combat healthcare corruption effectively motivates the present review.

The aim was thus to comprehensively collate the manifestations of different types of corruption in healthcare and keep abreast of any developments and trends. To systematically identify manifestations across various corruption types, we utilized the EU corruption typology and Thompson's institutional corruption framework [4, 35]. We chose the EU and Thompson typologies for specific reasons. The EU typology specifically caters to the healthcare sector rather than country characteristics (such as being a low, middle, or high-income country, [42]) and, in our opinion, offers a thorough and systematic guide for examining the different types of corruption. We consider the Thompson typology a valuable addition that enhances our understanding of corrupt behavior primarily associated with criminal activity. Table 1 provides an overview of the various types of corruption and the stakeholders involved, as delineated by the two typologies.

**Table 1:** Corruption typologies

	Type	Abbreviation	Exemplary manifestations	Stakeholders
EU [4]	Bribery in medical service delivery	BribMSD	Informal payments, in-kind gifts, absenteeism	Patients, providers
	Procurement corruption	ProcCorr	Customized tendering, kickbacks, favoritism, collusion	Industry, providers, regulators
	Improper marketing relations	ImproperMR	Gifts, provider sponsoring (conferences, continuing medical education), consultancy contracts	Industry, providers, regulators
	Misuse of high-level positions and networks	MisuseHPN	Lobbying, trading in influence, nepotism, fraternalism, favoritism	Industry, providers, regulators, political parties
	Undue reimbursement claims	UndueRC	Creative billing (upcoding), fraudulent billing	Providers, payers (governments, insurance)
	Fraud and embezzlement of medical drugs, devices and services	FraudDDS	Sale of (counterfeit) drugs for private gain	Providers
Thompson [35]	Institutional corruption	InstCorr	Behavior (not necessarily illegal) undermining an institution's primary purpose or fostering inappropriate dependencies	All potential stakeholders

The EU typology distinguishes six distinct corruption types, each contingent upon the sector and the involvement of specific stakeholders (patients, providers, industry, regulators, payers, and political parties). These types encompass bribery in medical service delivery (BribMSD), procurement corruption (ProcCorr), improper marketing relations (ImproperMR), misuse of high-level positions and networks (MisuseHPN), undue reimbursement claims (UndueRC), and fraud and embezzlement of medical drugs, devices and services (FraudDDS).

Table 1 highlights exemplary manifestations of each type of corruption, which are the focus of our review. In this study, a manifestation refers to any actual misconduct of individuals, organizations, or entire networks.

BribMSD primarily involves exchanging a financial advantage or non-cash gift between the provider and the patient for privileges or treatment. ProcCorr captures misconduct, such as (pharmaceutical/medical device) industry agents bribing doctors to procure medical drugs, and involves industry, providers, and regulators. ImproperMR can occur between industry, service providers, and regulators, encompassing gifts, sponsorship, and money-spinning consultancy contracts. MisuseHPN can develop among regulators, political parties, industry, and providers, manifesting, among others, as lobbying, favoritism, or trading in influence. UndueRC involves payers and service providers and comes as insurance fraud or more subtle acts, such as upcoding. Service providers engage in FraudDDS by harvesting healthcare funds for private gain [4].

Thompson [35] distinguishes individual corruption, prevalent in most corruption types according to the EU typology, from InstCorr based on three crucial elements: the gain for the institution, the advantage for the beneficiary outside the institution, and the interaction between the two. In InstCorr, the gain is a by-product of appropriate service. In individual corruption, the gain is not part of proper action. The second element concerns the advantage granted to the beneficiary outside the institution. Here, Thompson distinguishes between an undeserved advantage, implying individual corruption, and an advantage where the focus lies not on the beneficiary's worthiness but on the manner of bestowal. The third element relates to the connection between the gain and the benefit. While individual corruption often involves quid pro quo motives, InstCorr typically stems from systemic conditions and represents a pattern in regular service delivery.

The rest of the paper comprises four sections. Sect. 2 explains the methodology for this narrative review. Sect. 3 unfolds the results. Sect. 4 discusses the findings, navigates through the implications and limitations of our study and concludes with remarks reaffirming the value of our research.

## Materials and methods

We opted for a narrative literature review to identify as many different actual manifestations of corruption as possible across a broad spectrum of research spanning various areas and perspectives. While allowing a comprehensive perspective, considered particularly relevant for a highly complex phenomenon like corruption, a narrative review might suffer from biases due to subjective paper selection and interpretation. To overcome these limitations, we followed a rigorous protocol (see Literature search, Paper selection and Paper analysis Sections) for searching, selecting, and analyzing the original papers.

### Literature search

We selected the period from 2013 to 2022 to cover a decade and ensure up-to-date coverage of corrupt manifestations. The decision to commence the review in 2013 aligns with the rising scholarly attention towards InstCorr in the health sector.

We initially conducted a primary search for scientific journal articles on corruption in the healthcare sector using SCOPUS, which is known for its extensive collection of peer-reviewed articles across diverse disciplines. Additionally, we performed a supplementary search in EBSCO to ensure thorough identification of relevant articles for the review. The final queries for journal articles published in English that resulted from preceding test queries were (TITLE-ABS-KEY ( corruption) AND ABS (( medical AND health) OR (health AND care) OR healthcare OR (medical AND device*) OR pharma*)) AND PUBYEAR > 2012 AND PUBYEAR < 2023 AND (LIMIT-TO (SRCTYPE, "j")) AND (LIMIT-TO (PUBSTAGE, "final")) AND (LIMIT-TO (LANGUAGE, "English")) for SCOPUS and (TI corruption OR AB corruption OR SU corruption) AND (AB ( medical AND care) OR AB ( health AND care) OR AB ( medical AND device*) OR pharma*)) for EBSCO.

### Paper selection

**Fig. 1:**
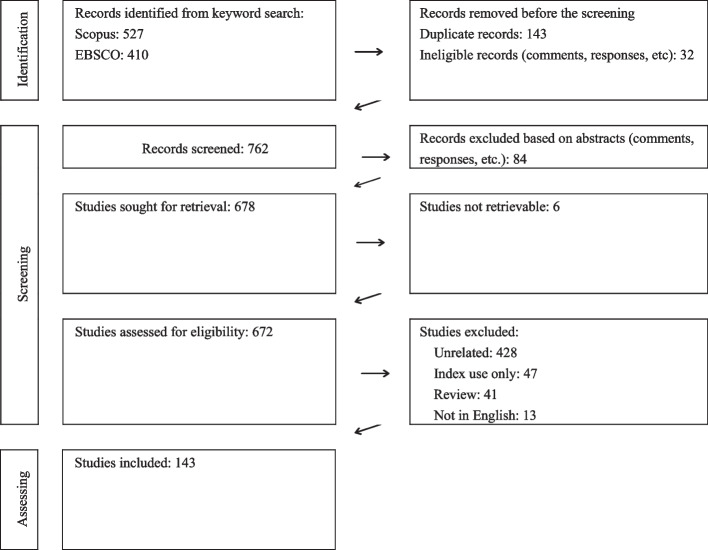
Paper selection

We used the Prisma scheme to document the selection of relevant papers [24] and ensure reproducibility (Fig. 1). The keyword search returned 937 records. After removing duplicates and evident short contributions (e.g. comments, editorials, etc.) based on the titles and page numbers, we screened the abstracts of the remaining 762 records, excluding an additional 84 ineligible contributions. We could not retrieve six articles from the remaining 678 papers, leading us to assess the eligibility of 672 documents.

We included only articles addressing actual manifestations of at least one of the seven corruption types. The exclusions encompassed articles that did not discuss corruption manifestations in the health sector ('Unrelated'), focused solely on corruption indices ('Index use only'), were review papers ('Review'), and were not in English despite our query excluding non-English language publications ('Not in English').

While the focus on the health sector explains the exclusion of corruption studies outside the health sector ('Unrelated'), we justify excluding index-use papers and reviews as follows. Index-use articles do not contribute to our research question, enhancing our understanding of how corruption manifests and evolves in the health sector. We did not consider prior reviews as they had a different scope or lacked up-to-date information. Moreover, we aimed to synthesize the literature independently to offer a fresh perspective on the dynamics of the manifestations of corruption.

Regarding InstCorr, we included papers that explicitly refer to the InstCorrr framework and those investigating institutional settings (without referencing InstCorr explicitly) that likely promote behavior that systemically compromises an overall purpose. However, categorizing the contributions along the two main typologies and even within the EU typology may occasionally be blurred following the overlap of corrupt activities.

The article selection process passed several stages, with the authors re-evaluating the remaining articles after each exclusion, resulting in 143 papers. A supplementary file contains the references and their identifier (ID). In the following sections, we refer to these references with their ID in curly brackets, i.e. {ID}, to distinguish them from the in-text references in square brackets covered in the reference list at the end of this paper.

### Paper analysis

We employed a template to analyze the selected original articles consistently. In addition to the seven possible types of corruption and their associated manifestations, this template covered further contextual information, such as standard bibliometric data, the aims of the article, the research questions, and the involved countries or regions and institutional sectors. We also collected information regarding the research approach (empirical or theoretical), the data sources (survey data, observational data, interview data, literature, newspapers, etc.), the study design (qualitative, quantitative, or mixed methods), and the specific methodological approach (thematic analysis, content analysis, regression analysis, etc.). In addition, we compiled the drivers and effects of corruption and actual and potential remedies, but these are not the topics of this article.

## Results

Table 2 displays the frequency of addressing corruption types, with bold diagonal numbers highlighting their occurrences. The total frequencies surpass the number of articles (143), reflecting that each article included in the review may address multiple types of corruption. BribMSD received the most analyses, followed by MisuseHPN and InstCorr. However, InstCorr comprises 12 articles from a 2013 special issue in the Journal of Law, Medicine, and Ethics [26]. Studies on ProcCorr and ImproperMR each amount to 29 articles, while 25 contributions cover FraudDDS. With 14 contributions, UndueRC received the slightest investigation.

**Table 2:** Frequency distribution of corruption types (*n* = 143)

	BribMSD	ProcCorr	ImproperMR	MisuseHPN	UndueRC	FraudDDS	InstCorr
BribMSD	**63**	9	9	17	8	14	1
ProcCorr		**29**	8	17	6	12	2
ImproperMR			**29**	14	5	7	12
MisuseHPN				**51**	10	17	13
UndueRC					**14**	8	1
FraudDDS						**25**	2
InstCorr							**38**

The numbers above the diagonal depict the frequency of studies exploring multiple corruption types. InstCorr frequently coincides with MisuseHPN (13) and ImproperMR (12), highlighting systemic misconduct in ImproperMR and MisuseHPN. Its occurrence in conjunction with other forms of corruption is rare, typically happening only once or twice.

The studies exhibit a significant geographic variation across the corruption types (Table 3). BribMSD studies are evenly spread across Africa, Asia, and Europe. The studies on BribMSD target the poorest countries in Africa and Asia [42], including Congo, Ethiopia, Rwanda, Sierra Leone, Uganda, and Afghanistan. In Europe, Ukraine—a country with a lower middle-income economy—frequently emerges in discussions on bribery. Cross-country analyses and studies without a specific country focus combined show a comparable frequency. However, many articles addressing ProcCorr, ImproperMR, and InstCorr have no country focus. Among those that have, Europe, particularly Ukraine (ProcCorr), Asia (ImproperMR), and North America, particularly the United States (InstCorr), emerge as predominant. Asian studies lead research regarding MisuseHPN, UndueRC, and FraudDDS.

**Table 3:** Geography, data, methods, stakeholders, and sectors

	BribMSD	ProcCorr	ImproperMR	MisuseHPN	UndueRC	FraudDDS	InstCorr
**Geography**							
Africa	13	4	–	6	2	5	–
Asia	16	5	8	16	7	7	3
Australia	–	–	1	1	–	–	1
Europe	17	8	4	8	1	3	2
North America	1	–	3	7	1	1	10
South America	1	2	–	1	–	–	–
Cross-country	10	–	3	2	–	4	5
NA	5	10	10	10	3	5	17
**Data**							
Literature/Reports/Others	+	++	+++	++	++	++	+++
Surveys/Interviews/FGD	+++	++	+	++	++	++	+
**Methods**							
Qualitative (Thematic)	++	+++	+++	+++	+++	+++	+++
Quantitative (Regression, Mixed Methods, Other)	++	+	+	+	+	+	+
**Stakeholders**							
Patients (Relatives, Friends)	x			x	x		
Healthcare Funders					x	x	
Healthcare Providers	x	x	x		x	x	x
Industry Representatives		x	x	x			x
Public Officials		x	x	x			x
Politicians		x		x			
Researchers			x	x			x
Medical Societies/Advisory Committees/Others			x				x
Patient Advocacy Groups			x				x
**Sectors**							
Academic			x	x			x
Health (services)	x	x	x	x	x	x	x
Insurance					x		
Pharmaceutical/Devices	x	x	x	x	x	x	x
Political		x	x	x			x
Regulatory			x	x			x

*NA* Not Applicable, *FGD *Focus Group Discussions

+++ Prominently/ ++ Evenly/ + Sparcely represented

Differences persist in the dominating data utilized across corruption types. BribMSD primarily draws upon (secondary) survey data (e.g. the Eurobarometer survey, life-in-transition survey, and global corruption barometer) and primary data gleaned from interviews and focus group discussions (FGD). Conversely, InstCorr studies heavily rely on secondary data from scholarly literature, case reports, legal documents, and other resources. Except for ImproperMR, where secondary data from relevant literature predominates over primary data from interviews and FGD and survey data, the data sources across the remaining corruption types (ProcCorr, MisuseHPN, UndueRC, FraudDDS) are balanced. Rare data sources comprise Twitter tweets {416}, mystery client visits {711}, tenders {595}, COI disclosure data {565}, social media posts {410}, lobbying disclosure data {453}, social security claims {348} and covert shopping {76}.

Thematic analysis is the primary methodological approach across all corruption types. However, in BribMSD, econometric approaches are nearly as prevalent as thematic analyses, which makes bribery studies unique, as quantitative analyses are far less common in other corruption studies.

Regarding stakeholder involvement, the comparison between the EU typology and the findings from the reviewed articles reveals no significant disparities, only refinements. Thompson's framework allows all stakeholders to engage in InstCorr. Stakeholder involvement in BribMSD, UndueRC, and FraudDDS involves the fewest stakeholders. BribMSD comprises doctors, nurses, and pharmacists on the supply side and patients, including their relatives and friends, on the demand side. UndueRC and FraudDDS and involve providers as perpetrators. However, UndueRC also encompasses patient misbehavior. In both types of corruption, healthcare funders (such as health insurance and governments) are the bribed stakeholders. Conversely, the remaining corruption types (ProcCorr, ImproperMR, MisuseHPN, and InstCorr) encompass a broader spectrum of stakeholders, with industry representatives and researchers playing a significant role, particularly in ImproperMR and InstCorr.

The sectors involved directly correlate with the stakeholders engaged. ImproperMR underscores the pharmaceutical industry's significant influence on healthcare, academia, and regulatory sectors. The observation that improper marketing is not primarily the misconduct of individual perpetrators but rather the misconduct of entire sectors points to systemic dysfunction. Comparing stakeholders and sectors of ImproperMR and InstCorr reinforces this conclusion. MisuseHPN demonstrates a similarly broad scope across sectors, while the remaining corruption studies (BribMSD, ProcCorr, UndueRC, FraudDDS) primarily concentrate on the health sector.

### Bribery in medical service delivery

BribMSD often comes in cash payments or gifts in kind (Table 4). Numerous studies {e.g. 62, 82, 92, 124, 141, 297, 299, 321, 416, 425, 656, 670, 711, 743} investigate informal payments without detailing them further. Others describe them regarding nature (cash versus non-cash benefits, {e.g. 6, 65, 105, 169, 265, 266, 267, 298, 664, 673, 710}), initiation (whether claimed by the provider or offered by the patient, {e.g. 274, 592}), timing (requested/provided before or after the treatment, {e.g. 123, 762}), and recipients (physician, pharmacist, nurses, other healthcare staff, {e.g. 229, 301, 408, 416, 631}). Patients bribe for shorter waiting times, (high-quality) treatment, or simply gratitude, but also for quite unusual purposes, such as falsifying true causes of death {82}, avoiding hospital staff withholding birth records {147}, and incentivizing discharge from or longer stays in the healthcare facility {631}.

**Table 4:** Bribery in medical service delivery (63 studies)

IDs	5, 6, 14, 17, 62, 65, 82, 92, 105, 123, 124, 141, 147, 169, 214, 229, 239, 265, 266, 267, 274, 297, 298, 299, 301, 311, 321, 357, 390, 408, 416, 425, 431, 435, 439, 472, 480, 490, 492, 516, 552, 561, 587, 590, 592, 594, 617, 631, 639, 640, 656, 664, 670, 673, 674, 696, 710, 711, 714, 721, 743, 758, 762
Manifestations	• Informal payments
	- No differentiation
	- Differentiation according to
	o type: cash vs. non-cash benefits
	o initiation: claimed by the provider vs. offered by the patient
	o timing: requested/provided before vs. after the treatment
	o recipient: physicians, pharmacists, nurses, other healthcare staff, cleaning staff, managers
	• Absenteeism and dual practice, including redirecting patients from public to private facilities
	• Nepotism

A considerable number of contributions addresses physician absenteeism and dual practice, including redirecting patients from public to private facilities to receive financial benefits {e.g. 5, 14, 147, 311, 431, 490, 516, 552, 639, 640, 714, 721, 758}. However, some studies also discuss nepotism as a specific type of misconduct {e.g. 239, 274, 590}.

### Procurement corruption

The papers scrutinize the misbehavior of the pharmaceutical industry, healthcare providers, public officials, and politicians in procuring pharmaceuticals, (sizeable) medical devices, and healthcare facility siting (Table 5). The primary form of corruption is usually bribery of stakeholders involved in the procurement process {e.g. 496, 603, 696}. Frequently, the industry acts as the bribing party, targeting public officials and members of tender committees. However, depending on the circumstances, bribery may also originate from other parties, such as state institutions, and may be aimed at the industry {82}. The industry pays bribes to influence tender processes, secure (public) procurement contracts, obtain licenses for constructing healthcare facilities, expedite contract procedures, win tenders, get drug and device registration, and manage customs clearance.

**Table 5:** Procurement corruption (29 studies)

IDs	61, 82, 101, 141, 179, 212, 216, 225, 229, 255, 296, 300, 370, 408, 431, 446, 496, 507, 529, 538, 592, 595, 603, 608, 617, 618, 639, 692, 696
Manifestations	• Bribery involving public officials, tender committees, providers, and pharmaceutical companies
	• Stakeholder collusion (including bid-rigging, abuse of public office, and distorting competition via satellite companies)
	• Price manipulation
	• Contract underperformance
	• Lobbying, favoritism, and fraternalism (i.e. awarding contracts to underqualified companies or those with ties to public officials or tender committee members)
	• Fraud, mishandling, and mismanagement of procurement funds
	• Further manifestations: Unduly influencing product selection and decision, improper tendering practices, arbitrary contractor selection, fraudulent and bogus offers, procuring unreliable/counterfeit/wrong-labeled/substandard/unregistered products, negotiating extra contracts for overdue projects, manipulating information (e.g. prices, maintenance needs, proper use of equipment), money laundering, monopolizing public contracts, breaching procurement contracts

Stakeholder collusion manifests as bid rigging {e.g. 82, 141, 216, 431, 595, 639, 696} (to set inflated prices) and establishment of satellite companies (to obscure tender participation and distort competition). When public authorities participate, ProcCorr manifests as an abuse of public office. Further manifestations encompass price manipulation, including overpricing {179, 216} and falsifying reference prices {446, 538, 603}, contract underperformance {179, 431, 639}, eventually due to awarding contracts to shell companies or inexperienced providers with ties to high-level officials {617}, lobbying {61}, and other forms of fraud at all stages of the procurement process {212, 370, 507, 529}. Manifestations also extend to information manipulation {538}, bogus offers {507}, and money laundering {603}. Several papers address ProcCorr in a disaster context. Most of them refer to the Severe Acute Respiratory Syndrome Coronavirus 2 (SARS-CoV-2) pandemic {212, 225, 529, 617, 692, 696}, highlighting fraud and mismanagement of medical equipment and funds, price collusion following revolving door conduct, procuring unreliable products, and bribery. A single contribution addresses breaches of procurement contracts during the Ebola outbreak in Africa {101}.

### Improper marketing relations

The studies identify various manifestations of ImproperMR, emphasizing its dynamics and illustrating the need for ongoing assessment of pertinent misconduct (Table 6). The industry's influence on doctors using money, gifts, and direct (e.g. covering travel costs) and indirect favors (e.g. supporting children's enrollment at the preferred school) to endorse off-label drug use and promote drug prescribing is widespread {145, 263, 408, 565, 635}. Various tactics for concealing improper payments emerge in this context, such as employing purported post-marketing surveillance studies {61}, falsified expense reports, offshore accounts, subsidiaries, slush funds, and sham contracts {226, 555}. ImproperMR includes deceptive, unethical, latent, or even criminal conduct, such as the systematic acculturation of medical experts as key opinion leaders (KOL) {43, 437, 145}, aggressive advertising of drugs and devices at healthcare facilities {17} and the suppression/delay/concealment of information about appropriate use and adverse effects of drugs and devices {44, 263}.

**Table 6:** Improper marketing relations (29 studies)

IDs	17, 43, 44, 106, 141, 145, 173, 226, 229, 263, 338, 408, 415, 416, 423, 431, 437, 446, 496, 552, 555, 565, 592, 596, 635, 639, 644, 700, 717
Manifestations	• Influencing/Incentivizing healthcare professionals with money, gifts, and personalized favors
	• Deceptive/unethical/latent/illegal conduct, including pro-active advertising of drugs and devices at healthcare facilities; suppressing/delaying/concealing information about appropriate use and adverse effects of drugs and devices; acculturating medical experts as KOL
	• Manipulating (pre-)clinical trial data, performing pseudo trials, capturing healthcare stakeholders via funding CME and clinical trials, exploiting informed consent and the rights of trial participants (especially in emerging countries), and engaging in ghostwriting and ghost management
	• Bribing (foreign) public officials
	• Capturing regulatory bodies, medical societies, and patient advocacy groups
	• Engaging in price manipulation (e.g. to obtain market authorization)
	• Promoting insurance fraud and manipulating reimbursement rules and Good Laboratory/Clinical/Medical Practice regulations
	• Committing fraud of public funds and programs

*CME* Continuing Medical Education, *KOL *Key Opinion Leaders

ImproperMR extends to a sector frequently involved in these corrupt links: the scientific arena. Studies highlight practices like concealing undesirable findings of clinical trials, eventually under the guise of data protection {44, 596}, systematically generating improper dependencies and COI via funding clinical trials, continuing medical education (CME) and medical expert committees, or initiating 'pseudo' trials designed for marketing purposes rather than scientific evidence {43, 44, 106, 141, 415, 431, 596, 639, 717}. The industry also captures prominent medical journals through these practices, threatening scientific and peer-review standards. A contribution that discusses advisory committee members' 'pay-later' COI {565, p. 17} resulting from the financial compensation of drug review committee members at some time after the decision-making highlights the dynamic nature of pertinent misconduct.

Further misconduct comprises bypassing statutory regulations on Good Laboratory/Clinical/Medical Practice, engaging in price manipulation {44, 145, 415, 496}, and influencing regulatory authorities and the state {717}, even across national borders. One contribution highlights misconduct that involves exploiting patients' rights, partly due to misunderstandings related to informed consent and insufficient adherence to trial standards, particularly notable in emerging countries {639}.

### Misuse of high-level positions and networks

MisuseHPN ranges from individuals' misbehavior to an entire industry's misconduct (Table 7). Frequent conduct comprises lobbying activities of the pharmaceutical industry to influence political decision-making processes and regulatory authorities, aiming at selectively enforcing or relaxing laws and regulations {105, 394, 431, 437, 483, 596}. Furthermore, the research underscores clientelism (e.g. appointing hospital managers based on political party affiliation rather than qualification {103}), nepotism (e.g. promoting family members or friends {141, 411, 565}), and favoritism (exemplified by politicians who disproportionately support their home regions {740}).

**Table 7:** Misuse of high-level positions and networks (51 studies)

IDs	82, 101, 103, 105, 106, 112, 141, 179, 200, 214, 225, 229, 252, 253, 288, 300, 318, 338, 347, 357, 370, 394, 408, 410, 411, 415, 416, 431, 436, 437, 453, 480, 483, 490, 496, 507, 538, 556, 582, 596, 608, 617, 635, 639, 656, 696, 700, 714, 721, 729, 740
Manifestations	• Lobbying (directed at governments, public officials, regulatory authorities, service providers, politicians, political parties, health insurance companies, governments, medical societies, and academic institutions)
	• Clientelism (oriented towards specific interest groups and high-level individuals)
	• Favoritism (i.e. preferring a person or a group over others) and nepotism/fraternalism (favoring relatives over others) across all sectors and stakeholders, including patients
	• (Cross-sector) collusion
	• Bribing high-level public officials and executive staff of health facilities and academic institutions
	• Abuse of authority

Further wrongdoing extends to collusion among stakeholders to divert money, such as International Federation of Red Cross and Red Crescent Societies staff colluding with bank officials to fraudulently access disaster funds {101}. Studies also highlight the bribing of high-level public officials to divert public funds for private or corporate gain {179} and controlling/manipulating/distorting information and facts, such as creating an ostensibly independent research foundation aimed at influencing (inter)national policies {318}. Furthermore, studies report on high-level providers intentionally provoking an emergency to create a teaching opportunity and senior staff abusing public resources for private gain and forcing subordinate staff to participate {721}. Researchers providing biased evidence, causing experts to recommend distorted treatment protocols, represents another misconduct {288}. In the SARS-CoV-2 pandemic context, a critical discourse highlights the conduct of reducing decision-making authority to a limited cadre of experts, fostering the possibility of abusing high-ranking positions {483}.

### Undue reimbursement claims

Dominant misconduct encompasses fraudulent and bogus reimbursement claims and improper practices like upcoding, unbundling, and overprovisioning services (Table 8). This misconduct has various forms, such as prescription drug-related fraud in the form of exaggerated billing of insurance companies {229}, private providers, whether hospitals or individual doctors, submitting exaggerated or bogus claims for treatments or tests {540, 608}, manipulating coding systems to inflate reimbursement by exaggerating the severity of a patient's condition {373, 552} and disregarding prescription standards {348}. The SARS-CoV-2 pandemic demonstrates additional instances, including medical professionals stockpiling medications ‒ purported as potential coronavirus treatments ‒ by prescribing them for personal use or their family members {696}.

**Table 8:** Undue reimbursement claims (14 studies)

IDs	105, 229, 348, 373, 408, 431, 436, 540, 552, 556, 608, 639, 696, 721
Manifestations	• Fraudulent claims (billing more/other services than provided)
	• Bogus claims (billing services not provided)
	• Improper claims (over-prescribing drugs/over-providing services, upcoding, unbundling)
	• Eligibility fraud, including stakeholders (patients, providers) manipulating information and identity theft
	• Physician-pharmacist collusion
	• Pharmacies disregarding prescription mandates
	• Duplicate registration of insurance members

Further misbehavior comprises patients hiding diseases at the time of buying insurance to avoid higher premiums {540}, physicians colluding with pharmacists to foster the mass sale of prescription drugs {408}, and enrolling patients multiple times in national health insurance to exploit double premiums {556}.

### Fraud and embezzlement of medical drugs, devices and services

Corrupt activities span various pertinent issues (Table 9). Commonly discussed misconduct covers embezzling, misappropriating, and misallocating funds, drugs, and devices {e.g. 141, 179, 200, 229, 357, 446, 561, 640}, and producing and circulating counterfeit or substandard medications (i.e. medications lacking proper or adequate active pharmaceutical ingredients, containing undisclosed substances, featuring incorrect dosages) and selling expired drugs after relabelling their expiry date {e.g. 44, 76, 236}. Misconduct extends to illicit drug trafficking, unauthorized storage and unregulated sale of drugs {408, 721}, and quantity fraud by documenting larger quantities than the patient received {105}. During disasters, additional and increased misconduct emerges, such as payroll fraud during the Ebola crisis{101}, issuance of fake Coronavirus Disease 2019 (COVID-19) test results {436}, and increased misappropriation of funds and medical equipment during the SARS-CoV-2 pandemic {212, 696}.

**Table 9:** Fraud and embezzlement of medical drugs, devices, and services (25 studies)

IDs	44, 76, 101, 105, 141, 179, 200, 212, 225, 229, 236, 357, 408, 431, 436, 446, 480, 561, 617, 639, 640, 696, 714, 721, 723
Manifestations	• Embezzling/Misappropriating/Misallocating health sector funds, medical drugs, and devices
	• Circulating counterfeit/substandard/expired medicines and supplies
	• Illegally producing/storing/selling (counterfeit/substandard/expired) drugs
	• Committing payroll fraud
	• Falsifying certificates and test results

### Institutional corruption

A subset of articles {116, 163, 164, 165, 173, 217, 222, 232, 257, 420, 437, 466, 611, 613, 654, and 662} explicitly engage with the works of Thompson or Lessig while the remaining articles do not directly reference the InstCorr framework. Instead, they delve into its core principles, such as disparities between an institution or system's intended purpose and the actual outcomes, COI impacting stakeholder performance, and improper dependencies among parties. One contribution addresses the 'institutional corruption' within healthcare bodies but uses the term independently of the InstCorr framework {268}.

Most research focuses on the pharmaceutical sector, leading to significant parallels with manifestations of ImproperMR. Table 10 elaborates on these manifestations, spanning research, marketing, and regulatory spheres, highlighting additional parallels with MisuseHPN and underscoring the intricate interdependencies among these entities.

**Table 10:** Institutional corruption (38 studies)

IDs	43, 61, 66, 106, 107, 116, 145, 163, 164, 165, 173, 200, 217, 222, 232, 252, 257, 263, 268, 288, 318, 338, 347, 410, 415, 418, 420, 423, 431, 437, 466, 483, 565, 596, 611, 613, 654, 662
Manifestations	• Cultivating financial COI in the scientific sector (researchers, publishers, editors, reviewers, academic institutions), regulatory authorities (FDA), medical expert groups, providers
	• Identifying and involving potential KOL through speaker fees, consulting fees, and future publication incentives, aiming to influence medical perspectives by endorsing statements at conferences, symposia (simultaneously shaping their focus), and CME lectures highlighting the benefits of new drugs
	• Biasing clinical trials:
	- Selection bias, such as cherry-picking trial subjects or excluding subjects from trial participation
	- Reporting bias (selective reporting), such as documenting something not covered in the initial research agenda, reporting trial results for a shorter than the entire trial period, reporting findings following narrowing the scope of the trial, suppressing unfavorable results, communicating results in misleading ways, substituting surrogate endpoints for actual clinical endpoints to make a drug look more efficacious, censoring trial results
	- Performance bias, such as providing ancillary treatments
	- Detection bias following ineffective blinding
	- Other biases, such as creating a trial environment lacking proper oversight due to the involvement of multiple stakeholders, including Contract Research Organisations (CRO), testing new drugs against placebos rather than established effective treatments and performing 'in-house' trials on their products, investing in duplicate patented medicines/devices with little therapeutic benefit and focusing on molecularly different but therapeutically similar drugs
	• Fostering the trial-journal pipeline, i.e. aligning trials with a drug's marketing objectives through on-demand publication, using 'publication planning' teams consisting of statisticians, researchers, ghostwriters, and journal editors who provide scientific support for the sponsor's drug
	• Widening diagnostic boundaries of illness (disease mongering) and fostering artificial grassroots initiatives (astroturfing) to introduce new products (instead of testing a scientific hypothesis)
	• Fostering inappropriate career dependencies through dependence research networks
	• Bullying independent researchers who arrive at unfavorable conclusions and denigrating critical (scientific) voices
	• Rule-making/trading in influence (lobbying and campaign finance) to foster the industry's interest (including market protection beyond patents, extending patent protection, increasing tax credits, reducing drug approval standards, maintaining the clinical trial data secrecy, avoiding price controls, reducing regulatory oversight of corporations and regulation bodies)
	• Rule-gaming (such as dividing donations across different branches of political parties at both state and federal levels to fall below mandatory disclosure thresholds and providing retrospective compensation to members of drug advisory committees)
	• Promoting non-disclosure of COI in industry-sponsored research, publications, and clinical practice guidelines
	• Counseling service providers in rule-gaming, such as designing remuneration transactions that meet their letter but bypass their intent
	• Exploiting asymmetric information in business-to-consumer and business-to-business marketing
	• Colluding with public institutions (e.g. CDC, Japan Tobacco) to shape scientific evidence

*COI *Conflicts of Interest, *FDA *Food and Drug Administration, *KOL *Key Opinion Leaders, *CME *Continuing Medical Education, *CDC *Centers for Disease Control and Prevention

A dominant form of industry misconduct spanning all areas involves nurturing financial COI {e.g. 145, 222, 257, 338, 420, 437, 466, 613}, thereby fostering inappropriate financial dependencies of the involved stakeholders, to shape their behavior in a manner that aligns with the industry's interests. Such undue dependencies may arise among any party with financial ties to the industry, whether stemming from CME funding {654}, financially supporting medical societies {163}, user-fee funding of regulatory agencies (such as the Food and Drug Administration) {420} or financial incentives for providers to participate in marketing studies for non-interventional post-operative treatments, promoting off-label drug use and increasing prescriptions {466}. These dependencies also manifest through advertisements in scientific journals {596}.

Prominent misconduct affecting the realms of research and service delivery, partially intersecting with ImproperMR, involves the systematic acquisition of KOL. Such conduct entails identifying esteemed researchers, mapping their influence, and consistently managing them to advance industry interests by shaping scientific knowledge and influencing how healthcare providers diagnose and treat medical conditions {e.g. 145, 232, 418, 596, 654}.

Research also underscores biasing clinical trials as common misconduct (partly due to dependence corruption). This wrongdoing manifests in several ways, including selective enrollment of trial participants, such as excluding pre-trial participants already on medication experiencing significant side effects {288}, selective reporting of trial outcomes, like communicating findings other than the intended ones after failing to find the expected results {217} and biased trial conduct, such as encouraging researchers to administer medications to mitigate side effects of the tested drug {288}. Further biases arise from practices like conducting 'in-house' studies {596}, performing drug tests against placebos {420}, and researching only minor variations of existing drugs rather than investing in new ones to favor shareholders and investors at the expense of patients {222, 232}.

Promoting the trial-journal pipeline to influence the entire research process, starting with the design of the clinical trial to the publication of the outcome {420}, disease-mongering {163} astroturfing {596}, exploiting individual researchers' dependence on (industry-funded) networks {257} and harassing researchers to discourage negative conclusions {232} represent further wrongdoing. However, the contributions acknowledge that learned behaviors such as trust in company data or replicating improper actions and inappropriate incentives within scholars' career paths, compounded by the consequences of inadequate public research funding and the failure of publishers, editors, and reviewers to uphold standards of science and peer review, can indeed foster significant misconduct {e.g. 43, 116, 338}.

Rule-making and rule-gaming illustrate the industry's capture of regulatory authorities, thereby highlighting the considerable overlap between the manifestations of InstCorr and MisuseHPN. Lobbying, campaign contributions, and other types of financial relationships, including the industry's user-fee funding or lagged compensation of treatment advisory committee members {116, 420, 565}, reinforce the industry's ability to assert its various interests effectively.

Some contributions underscore further misconduct, such as non-disclosure of COI in industry-sponsored research, publications, and clinical practice guidelines{411}, counseling service providers to structure remuneration transactions to appear compliant while circumventing their true intent {611}, leveraging asymmetric information in business-to-business and business-to-consumer marketing {613} and colluding with public institutions (e.g. Centers for Disease Control and Prevention, Japan Tobacco {318, 410}).

## Discussion

What insights do we gain from the review? First, it highlights differences in addressing the individual types of corruption in academic discourse. The scientific literature predominantly emphasizes BribeMSD and the MisuseHPN. Articles on InstCorr also occupy a significant place, while ProcCorr, ImproperMR, and FraudDDS are moderately prevalent. UndueRC receives limited attention in the scientific literature. However, the divergent coverage of these corruption types could stem from database selection and corruption type-specific characteristics, such as pre-existing countermeasures and difficulties in identifying relevant misconduct, underscoring the need for further investigation into these areas.

Additionally, the disparity in how these corruption types are covered likely hinges on several factors: the problem's urgency, the availability and accessibility of relevant data, the public interest that regional or nationwide scandals can amplify, and the focus of national and international policy, often influenced by geographic relevance. Additionally, academic priorities, driven by funding bodies, play a crucial role in shaping the research agenda. Consequently, further research is needed to gain a balanced and comprehensive understanding of the contextual factors that influence the focus on different types of corruption in the literature.

Second, the review emphasizes the geographic variation across the corruption types. BribMSD poses a significant challenge to African, Asian, and post-socialist European healthcare systems. In low and lower-middle-income African and Asian countries, individuals financially incapable of bribing healthcare providers risk denial of essential healthcare services. The persistent prevalence of BribMSD in post-socialist countries results from the prevailing attitudes of the population towards corruption and their perceptions of what constitutes corrupt behavior. In those countries, bribes for health workers, especially doctors, are intended to express appreciation and ensure faster and supposedly higher quality treatment. Dual practice and absenteeism of medical staff also present significant challenges. In low and lower-middle-income countries, however, these manifestations can be life-threatening if patients lack access to medical care. In affluent, well-developed nations with robust essential healthcare services, the adverse effects of dual practice may not pose life-threatening risks but are still decidedly disadvantageous for the overall health system whenever providers' financial objectives compromise the health system's overarching goals, including equal access for equal needs. The review, therefore, highlights that both the severity of repercussions and the classification of corrupt behavior will likely depend on the context.

ProcCorr, another breeding ground for corruption, implies a considerable waste of money. Investigating ProcCorr is challenging because of highly complex procurement processes, limited oversight and access to relevant information, and sophisticated cover-ups. As with UndueRC, studies highlight data-based tools such as electronic procurement that help ensure procurement procedures are less susceptible to corruption. Additionally, many countries have adequate laws and regulations to combat ProcCorr. The OECD [23] provides an overview of applicable laws, rules, and tools, including general anti-bribery laws, laws tailored to procurement, initiatives that foster transparency in the procurement process, and tools that reduce information asymmetries and increase stakeholder participation. However, anti-corruption laws, whether broad or specific, are no guarantee for reducing corruption per se, as several studies and experiences from different countries show [11, 20, 37]. A wide range of factors can influence the effectiveness of laws. Laws require unambiguous wording, comprehensive problem coverage, appropriate sanctions, and independence from lobbyists' rule-making attempts. Laws also require consistent enforcement and foresight regarding potential rule-gaming, such as exploiting loopholes or the lack of independent jurisdiction and resources. With rule-making and rule-gaming as typical manifestations of InstCorr [28], the latter can potentially affect the effectiveness of legal approaches to curb individual corruption.

Additionally, anti-corruption legislation and compliance with the rules require regular evaluation to keep pace with corruption's dynamic nature and assess its impact on reducing corruption. Overall, it appears essential to complement legal initiatives with additional measures, such as transparency, oversight, awareness, and economic initiatives. However, a considerable challenge lies in addressing the complexity of corruption while maintaining a consistent and effective countermeasures system.

Employing two distinct corruption typologies exposes the interrelated nature of corrupt activities. This association becomes particularly apparent in the EU classifications of ImproperMR and MisuseHPN when juxtaposed with Thompson's InstCorr. These three corruption types unveil an intricate network involving many stakeholders, including the pharmaceutical industry, academia, regulators, medical advisory committees, and patient advocacy groups, giving rise to many potential corruption drivers. However, the InstCorr framework highlights that individuals seeking personal enrichment are not the sole drivers of corruption. Instead, structural elements rooted in institutions' motivational and incentive structures can foster COI and dependence corruption among multiple stakeholders. Therefore, blaming and penalizing individual wrongdoers may not be an appropriate solution. Instead, the InstCorr research recommends identifying, evaluating, and adjusting institutional frameworks that encourage such misbehavior.

Less frequently addressed than other corrupt practices, studies handle UndueRC. However, many countries with well-established health insurance systems have sophisticated plausibility checks in often automated insurance claims billing. Those make it easier to detect reimbursement fraud, such as billing for services not rendered or multiple billing for the same service. In developing countries where health insurance systems are still evolving, the distinction between UndueRC and BribMSD can blur, for example, when doctors bill patients for services covered by health insurance while simultaneously pocketing the insurance payments privately. Such behavior might explain ‒ at least partly ‒ the comparably high number of bribery studies in emerging Asian and African countries. However, sophisticated insurance systems face more significant problems resulting from doctors' rooms for maneuvers. Unbundling and upcoding, in particular, are issues here. These activities are challenging but not impossible to identify. Whenever systemic, but not criminal, misconduct appears, such behavior likely indicates InstCorr. Data mining approaches are promising tools for identifying such trends, but only a few studies examine the effectiveness of these approaches in uncovering relevant misconduct.

Within the EU category, FraudDDS includes misconduct not addressed by other forms of corruption. SARS-CoV-2 research highlights various fraudulent acts, such as national interests taking precedence over a globally coordinated disaster response, threatening entire populations. Literature also examines the dynamics and rapid occurrence of misconduct following exogenous shocks. Increased vulnerability to corruption during a pandemic sometimes arises from regulators' need for swift action, emphasizing the importance of preventive measures and informed remedial actions. Overall, evidence on pandemic-related fraud underscores the necessity of organizational changes to enable healthcare systems to absorb adverse impacts, adapt to new environments, and transform the health system towards functionality beyond the pre-shock level [1].

The InstCorr literature offers new perspectives on corrupt behavior and thoroughly examines its manifestations. The studies highlight the challenges in establishing a definitive basis for judging whether institutionally corrupt behavior has occurred. Somewhat, these acts veer away from a normative standard of behavior dictated by ethical considerations or fiduciary obligations but not criminal law. Academic literature also teaches us that it is insufficient to focus solely on individuals in the fight against corruption. Instead, it is crucial to scrutinize the environment and its influence on the behavior of those involved and to identify any distorted incentives. However, this approach can quickly become intricate and perplexing, requiring a focus on the misbehavior that is most likely to occur and has a significant detrimental impact. Although high-income countries primarily tackle InstCorr, low- and lower-middle-income countries can benefit from a comprehensive understanding of its manifestations. This knowledge allows political decision-makers in these countries to identify potential forms of corruption early and combat them effectively.

Third, synthesizing extensive manifestations of corruption in the health sector provides critical insights for policymakers to derive effective and robust anti-corruption measures at local and global levels. Attempting to eradicate corruption, however, is doomed to failure. Instead, we should focus on curbing corruption to cushion its adverse effects. In certain instances, like UndueRC and FraudDDS, robust anti-corruption laws can contribute to a lasting reduction in corruption, provided the relevant laws are consistently enforced. However, the InstCorr literature highlights that a legal framework has limitations in regulating systemic forms of misconduct that may not be inherently illegal, underscoring the necessity for organizational remedies.

Effective countermeasures must extend beyond the health sector to encompass other domains like education, legislation, international affairs, and public finance, thus complicating the situation further. Given the distinct responsibilities inherent in these sectors, divergent interests arise, adding complexity to coordinated efforts. Furthermore, confining remedial actions to local initiatives may prove inadequate following conflicting country-specific laws and regulations, divergent interpretations of corruption, and variations in the capacities of individual states to enforce countermeasures. Concerted cross-country research involving stakeholders from affected sectors is required to derive effective remedies. Assessing the effectiveness of existing and proposed countermeasures in light of the intricate nature of corruption highlights a promising research agenda.

The fight against corruption remains a Sisyphean task. Metaphorically speaking, while extinguishing one fire, another one flares up elsewhere. Vigilant surveillance of corrupt practices, encompassing the discernment of patterns and trends, is therefore pivotal. The wealth of scholarly insights into corrupt practices within healthcare and allied domains provides a solid groundwork for this endeavor.

Fourth, the review highlights the increased vulnerability of ProcCorr, MisuseHNP, and FraudDDS during pandemics, underscoring the need for preventative measures. Context-dependent variations in the effects of corruption also became apparent, enabling the development of countermeasures.

Finally, the review accentuates the significant contribution of the InstCorr literature in enhancing comprehension of ImproperMR and MisuseHPN while emphasizing the pivotal role of the scientific community in addressing these issues, thus signaling a crucial area necessitating further investigation.

While this review offers valuable insights into corruption within the health sector, it is crucial to acknowledge certain associated limitations.

First, the choices in selecting databases, keywords, and observation periods may introduce biases in the results. While we do not expect any significant impact from searching additional databases, we assume that changing (the combination of) keywords will influence the number of records identified. The advanced search for the keywords we selected confirms this assessment: In the 2013 Special Issue on InstCorr in the Journal of Law, Medicine, and Ethics, the search did not retrieve all articles of the special issue—despite their potential relevance—because their titles, abstracts, subjects or specified keywords did not match our search terms. As this is a limitation independent of the type of corruption, we decided not to include these articles to avoid biasing the selection process towards InstCorr. However, we see this limitation as an impetus for further research using broader search strategies, such as bibliographic snowballing, to capture additional manifestations of (institutional) corruption.

Second, categorizing contributions along the corruption typologies and their types is susceptible to subjectivity, further complicated by the interconnected nature of corrupt activities. Third, focusing solely on the health sector may pose challenges in identifying emerging forms of corruption from other industries that could infiltrate the health sector. Nonetheless, these limitations serve as an impetus for future research endeavors.

Overall, we believe the central role of the review, highlighting manifold manifestations, lies in its relevance to combating corruption effectively. Understanding corrupt behavior's diverse and often unpredictable manifestations is not merely an academic exercise but a fundamental necessity in devising robust and effective anti-corruption strategies. Theoretical models and considerations may fall short as they fail to fully represent or capture misbehavior's complex and dynamic nature as it manifests in reality. By constantly updating the relevant knowledge through detailed reviews of how corruption manifests and develops, we equip researchers, policymakers, law enforcement, and regulatory bodies with starting points for actions.

However, we recognize that documentation alone is insufficient. Knowledge about misconduct has to lead to identifying who is involved in misbehavior, under what circumstances, how often and why misconduct occurs, and how serious it is. Therefore, understanding misbehavior is at the core of further action: identifying risk factors, estimating impacts, and crafting effective anti-corruption strategies. Thus, while uncovering manifestations is a critical first step, it is only the beginning of a deeper exploration into the dynamics of corrupt practices, which should be the focus of future research agendas.

### Supplementary Information

Supplementary Material 1.

## Data Availability

No datasets were generated or analysed during the current study.

## Abbreviations

BribMSD: Bribery in medical service delivery
ProcCorr: Procurement corruption
ImproperMR: Improper marketing relations
MisuseHPN: Misuse of high-level positions and networks
UndueRC: Undue reimbursement claims
FraudDDS: Fraud and embezzlement of medical drugs, devices and services
InstCorr: Institutional corruption
COI: Conflicts of Interest
FDA: Food and Drug Administration
KOL: Key Opinion Leaders
CME: Continuing Medical Education
CDC: Centers for Disease Control and Prevention
OECD: Organisation for Economic Co-operation and Development
EU: European Union

## References

[1] Behrens D, Rauner M, Sommersguter-Reichmann M (2022). Why resilience in health care systems is more than coping with disasters: implications for health care policy. Schmalenbach J Bus Res.

[2] Cevik S, Jalles J, Corruption kills: global evidence from natural disasters 2023. International monetary fund, https://pure.port.ac.uk/ws/portalfiles/portal/17778625/The_Financial_Cost_of_Healthcare_Fraud_Report_2014_11.3.14a.pdf. Accessed 9 July 2024.

[3] Dincer O, Teoman O (2019). Does corruption kill? Evidence from half a century infant mortality data. Soc Sci Med.

[4] European Commission, Study on corruption in the healthcare sector 2013. European Union, https://ec.europa.eu/home-affairs/sites/homeaffairs/files/what-is-new/news/news/docs/20131219_study_on_corruption_in_the_healthcare_sector_en.pdf (4 August 2017, date last accessed).

[5] European Commission 2017, Updated study on corruption in the healthcare sector, European Union, https://ec.europa.eu/home-affairs/sites/homeaffairs/files/20170928_study_on_healthcare_corruption_en.pdf.

[6] European Commission, Corruption 2023, https://home-affairs.ec.europa.eu/policies/internal-security/corruption_en (10 July 2023, date last accessed).

[7] European Healthcare Fraud & Corruption Network, Waste Typology Matrix 2023, https://www.ehfcn.org/what-is-fraud/ehfcn-waste-typology-matrix/ (13 July 2023, date last accessed).

[8] Farzanegan M, Hofmann H (2021). Effect of public corruption on the COVID-19 immunization progress. Sci Rep.

[9] Gee J, Button M, The financial cost of healthcare fraud 2014. PKF Littlejohn LLP and University of Portsmouth, https://pure.port.ac.uk/ws/portalfiles/portal/17778625/The_Financial_Cost_of_Healthcare_Fraud_Report_2014_11.3.14a.pdf. Accessed 9 July 2024.

[10] Hanf M (2011). Corruption kills: estimating the global impact of corruption on children deaths. PLoS ONE.

[11] Hussmann K, Anti-corruption policy making in practice: what can be learned for implementing Article 5 of UNCAC? 2007. U4 Anti-Corruption Resource Center, https://www.u4.no/publications/anti-corruption-policy-making-in-practice-whatcan-be-learned-for-implementing-article-5-of-uncac. Accessed 9 July 2024.

[12] Ionescu L (2018). Where does it hurt? Governance and corruption in health care delivery in CEE countries. Econ Manag Financ Mark.

[13] Kabia E (2021). The hidden financial burden of healthcare: a systematic literature review of informal payments in Sub-Saharan Africa. Wellcome Open Res.

[14] Karunamoorthi K (2014). The counterfeit anti-malarial is a crime against humanity: a systematic review of the scientific evidence. Malaria J.

[15] Khan A, Abedin S, Rahman M, Khan S (2022). Effects of corruption and income inequality on the reported number of COVID-19 cases and deaths: evidence from a time series cross-sectional data analysis. PLOS Glob Public Health.

[16] Kohler J, Dimancesco D (2020). The risk of corruption in public pharmaceutical procurement: how anti-corruption, transparency and accountability measures may reduce this risk. Glob Health Action.

[17] Kuzmenko O (2020). Features of the investigation of corruption abuses in the medical industry. Sys Rev Pharm.

[18] Lessig L (2013). Foreword: 'Institutional corruption' defined. J Law Med Ethics.

[19] Mostert S (2015). Corruption in healthcare systems and its effect on cancer care in Africa. Lancet Oncol.

[20] Mungiu-Pippidi A, Dadašov R (2017). When do anti-corruption laws matter? The evidence on public integrity enabling contexts. Crime Law Soc Change.

[21] Naher N (2020). The influence of corruption and governance in the delivery of frontline health care services in the public sector: a scoping review of current and future prospects in low and middle-income countries of south and southeast Asia. BMC Public Health.

[22] Novak A (2020). Anti-corruption policy under the conditions of overcoming the consequences of the coronavirus pandemic. Sys Rev Pharm.

[23] OECD, Preventing corruption in public procurement 2016, https://www.oecd.org/gov/ethics/Corruption-Public-Procurement-Brochure.pdf (4 December 2023, date last accessed).

[24] Page M (2021). The PRISMA 2020 statement: an updated guideline for reporting systematic reviews. Brit Med J.

[25] Pieterse P, Lodge T (2015). When free healthcare is not free. Corruption and mistrust in Sierra Leone's primary healthcare system immediately prior to the Ebola outbreak. Int Health..

[26] Rodwin M (2013). Institutional corruption and the pharmaceutical industry. J Law Med Ethics..

[27] Saeed G, Kohler J, Cuomo R, Mackey T (2022). A systematic review of digital technology and innovation and its potential to address anti-corruption, transparency, and accountability in the pharmaceutical supply chain. Expert Opin Drug Saf.

[28] Salter M. Lawful but corrupt: gaming and the problem of institutional corruption in the private sector 2010. Harvard Business School. https://www.hbs.edu/faculty/Publication%20Files/11-060.pdf. Accessed 9 July 2024.

[29] Sánchez-Duque J (2021). The ignored pandemic of public health corruption: a call for action amid and beyond SARS-COV-2/COVID-19. J Exp Biol Agric Sci.

[30] Savedoff W, Hussmann K. Why are health systems prone to corruption?, in: Global Corruption Report 2006, ed. by Transparency International. Pluto Press; 2006. p. 4–16.

[31] Sharma S, Singhal S, Tarp F (2021). Corruption and mental health: evidence from Vietnam. J Econ Behav Organ.

[32] Shepler S (2017). 'We know who is eating the ebola money!': corruption, the state, and the ebola response. Anthropol Q.

[33] Sommersguter-Reichmann M (2018). Individual and institutional corruption in European and US healthcare: overview and link of various corruption typologies. Appl Health Econ Health Policy.

[34] Thompson D (1993). Understanding financial conflicts of interest. N Engl J Med.

[35] Thompson D. Ethics in Congress. From individual to institutional corruption. Washington, DC: Brookings Inst; 1995.

[36] Thompson D. Two concepts of corruption 2013a. Edmond J Safra Center for Ethics. http://ethics.harvard.edu/workingpapers-series. Accessed 17 July 2023.

[37] Thompson K (2013). Does anti-corruption legislation work?. Int Trade Bus L Rev.

[38] Transparency International, Our national chapters 2023a, https://www.transparency.org/en/our-national-chapters (13 July 2023, date last accessed).

[39] Transparency International, What is corruption? 2023b, https://www.transparency.org/en/what-is-corruption (10 August 2022, date last accessed).

[40] Vian T (2008). Review of corruption in the health sector: theory, methods and interventions. Health Policy Plann.

[41] Vian T, Agnew B, McInnes K (2022). Whistleblowing as an anti-corruption strategy in health and pharmaceutical organisations in low- and middle-income countries: a scoping review. Glob Health Action.

[42] World Bank, World Bank Country and Lending Groups 2024, https://datahelpdesk.worldbank.org/ (2 April 2024, date last accessed).

[43] Zhang Y (2022). The relationship between corruption perception and depression: a multiple mediation model. Psychol Res Behav Manag.

