# Supplementary material for: Untangling the corruption maze: exploring the complexity of corruption in the health sector
Source: Health Econ Rev. 2024 Jul 12;14:50. doi: 10.1186/s13561-024-00530-6 (PMC11241952; doi:10.1186/s13561-024-00530-6)
Supplement: Supplementary file 1 — Supplementary Material 1. [file 13561_2024_530_MOESM1_ESM.docx]

Supplementary File: Review References

| ID | Reference |
| --- | --- |
| 5 | Abdallah W, Chowdhury S, Iqbal K. Access and fees in public health care services for the poor: Bangladesh as a case study. Oxf Dev Stud 2022;50:209–24. |
| 6 | Aboutorabi A, et al. Factors affecting the informal payments in public and teaching hospitals. Med J Islam Repub Iran 2016;30:315. |
| 14 | Agwu P, et al. Private money-making indulgence and inefficiency of primary healthcare in Nigeria: a qualitative study of health workers’ absenteeism. Int J Public Health 2020;65:1019–26. |
| 17 | Ahmed F, et al. Key challenges to optimal therapeutic coverage and maternal utilisation of CMAM program in rural Southern Pakistan: a qualitative exploratory study. Nutrients 2022;14. |
| 43 | Amsterdam J, McHenry L, Jureidini J. Industry-corrupted psychiatric trials. Psychiatr Pol 2017;51:993–1008. |
| 44 | Amudha R, Alamelu R, Cresenta Shakila Motha L, Badrinath V. Pharma advertising - Is it deceptive? Res J Pharm Biol Chem Sci 2015;6:1158–67. |
| 61 | Badawi D, Alkhamis Y, Qaddoumi M, Behbehani K. National transparency assessment of Kuwait’s pharmaceutical sector. Health Policy 2015;119:1275–83. |
| 62 | Badun M. Determinants of disability pensions in Croatia: the role of institutions. Public Sect Econ 2017;41. |
| 65 | Baji P, Pavlova M, Gulácsi L, Groot W. Exploring consumers’ attitudes towards informal patient payments using the combined method of cluster and multinomial regression analysis - the case of Hungary. BMC Health Serv Res 2013;13:1–14. |
| 66 | Baker D. The Future of the Pharmaceutical Industry: Beyond Government-Granted Monopolies. J Law Med Ethics 2021;49:25–9. |
| 76 | Bate R, Mathur A. Corruption and Medicine Quality in Latin America: A Pilot Study. BE J Econ Anal Policy 2018;18:20170076. |
| 82 | Batyrgareieva V, Babenko A, Kaija S. Corruption in medical sphere of Ukraine: current situation and ways of prevention. Wiad Lek 2019;72:1814–21. |
| 92 | Binyaruka P, et al. Supply-side factors influencing informal payment for healthcare services in Tanzania. Health Policy Plann 2021;36:1036–44. |
| 101 | Boateng O. Biological citizenship through litigation: Ebola survivors in Sierra Leone and the suit to redefine corruption. Crit Public Health 2022;32:747–58. |
| 103 | Bolton S, Charalampopoulos V, Skountridaki L. Selective consent and dissent: professional response to reform in the post-crisis Greek NHS. Work Employ Soc 2019;33:262–79. |
| 105 | Borooah V. Issues in the provision of health care in India: an overview. Arthaniti J Econ Theory Practice 2022;21:43–64. |
| 106 | Borsook D, Bernat J. Headache professional societies: ethical challenges and suggested solutions. Headache 2017;57:1273–83. |
| 107 | Borujeni H, Ostovar R, Soltani H, Jafarpourm H. Presenting a model for the treatment of systemic (organisational) illnesses in social security hospitals of Isfahan. Arch Pharm Pract 2019;10:91–105. |
| 112 | Bressan T, et al. Challenges of design, implementation, acceptability, and potential for, biomedical technologies in the Peruvian Amazon. Int J Equity Health 2022;21:183. |
| 116 | Brown A. Understanding pharmaceutical research manipulation in the context of accounting manipulation. J Law Med Ethics 2013;41:611–9. |
| 123 | Burai P. ‘One does everything to make life better.’ Petty corruption and its legal implications in Hungary. Comp Southeast Eur Studies 2018;66:349–70. |
| 124 | Burcea M, Toma S, Papuc R. Patients’ satisfaction with the health care system in the age of globalisation: the case of Romania. Transylvanian Rev Adm Sci 2014;Special Issue:5–20. |
| 141 | Chattopadhyay S. Corruption in healthcare and medicine: why should physicians and bioethicists care and what should they do? Indian J Med Ethics 2013;10:153–9. |
| 145 | Chen X. Pharmaceutical sales representatives in the United States and China: the need for professional public space. Health Care Anal 2022;30:35–56. |
| 147 | Choguya N. Corruption in health service delivery: the case of maternal health in rural Zimbabwe. Rev Hum Factor Stud 2018;24:81–104. |
| 163 | Cosgrove L, Peters S, Vaswani A, Karter J. Institutional corruption in psychiatry: case analyses and solutions for reform. Soc Pers Psychol Compass 2018;12:1. |
| 164 | Cosgrove L, Wheeler E. Drug firms, the codification of diagnostic categories, and bias in clinical guidelines. J Law Med Ethics 2013a;41:644–53. |
| 165 | Cosgrove L, Wheeler E. Industry’s colonisation of psychiatry: ethical and practical implications of financial conflicts of interest in the DSM-5. Fem Psychol 2013b;23:93–106. |
| 169 | Dallera G, Palladino R, Filippidis F. Corruption in health care systems: trends in informal payments across twenty-eight EU countries, 2013-19. Health Affairs 2022;41:1342–52. |
| 173 | David-Barrett E, Yakis-Douglas B, Moss-Cowan A, Nguyen Y. A bitter pill? Institutional corruption and the challenge of antibribery compliance in the pharmaceutical sector. J Manag Inq 2017;26:326–47. |
| 179 | Della Porta D, Sberna S, Vannucci A. Bribery in healthcare: the organisation of political corruption in the healthcare sector. Politiche Soc 2015;2:227–45. |
| 200 | Egharevba E, Atkinson J. The role of corruption and unethical behaviour in precluding the placement of industry sponsored clinical trials in sub-Saharan Africa: Stakeholder views. Contemp Clin Trials Commun 2016;3:102–10. |
| 212 | Eze O, Ajah B, Nwonovo O, Atama C. Health sector corruption and COVID-19 outbreak: evidence from Anambra and Enugu States, Nigeria. J Contemp Afr Stud 2022;40:34–46. |
| 214 | Fagan A, Sircar I. Transformation all the way down? European Union integration and the professional socialisation of municipal health officials in Serbia. J Common Mark Stud 2020;58:688–705. |
| 216 | Fedotova H, Kosilova O, Maryna Kobets M. Protection of life and health of citizens in the sphere of production and circulation of medicines and medical products: a legal aspect. J Hist Cult Art Res 2020;9:30. |
| 217 | Feldman Y, Gauthier R, Sehuler T. Curbing misconduct in the pharmaceutical industry: insights from behavioral eEthics and the behavioral approach to law. J Law Med Ethics 2013;41:620–8. |
| 222 | Fields G. Parallel problems: Applying institutional corruption analysis of Congress to big pharma. J Law Med Ethics 2013;41:556–60. |
| 225 | Forman L, Kohler J. Global health and human rights in the time of COVID-19: response, restrictions, and legitimacy. J Hum Rights 2020;19:547–56. |
| 226 | Fox T. Health care under the FCPA: lessons learned and going forward. J Health Care Compliance 2018;20:27–34. |
| 229 | Frolova I, Zakirova L, Khamitova L. Results of research of corruption’s problem in the health care system of Tatarstan. Life Sci J 2014;11:398–402. |
| 232 | Gagnon M. Corruption of pharmaceutical markets: addressing the misalignment of financial incentives and public health. J Law Med Ethics 2013;41:571–80. |
| 236 | Ganor B, Halperin Wernli M. The infiltration of terrorist organisations into the pharmaceutical industry: Hezbollah as a case study. Stud Confl Terror 2013;36:699–712. |
| 239 | Getachew T, et al. Health extension workers’ perceived health system context and health post preparedness to provide services: a cross-sectional study in four Ethiopian regions. BMJ Open 2021;11:e048517. |
| 252 | Gore R. Ensuring the ordinary: politics and public service in municipal primary care in India. Soc Sci Med 2021;283:114124. |
| 253 | Gorodensky A, Kohler J. State capture through indemnification demands? Effects on equity in the global distribution of COVID-19 vaccines. J Pharm Policy Pract 2022;15:50. |
| 255 | Gottschalk P. Application of the theory of convenience to a case of illegal public procurement of healthcare services. Int J Procure Manag 2019;12:621. |
| 257 | Gray G. The ethics of pharmaceutical research funding: a social organisation approach. J Law Med Ethics 2013;41:629–34. |
| 263 | Gutorova N, Soloviov O, Olejnik D. Improper healthcare marketing: German and Ukrainian experience in prevention. Wiad Lek 2019;72:2404–9. |
| 265 | Habibov N. Effect of corruption on healthcare satisfaction in post-soviet nations: a cross-country instrumental variable analysis of twelve countries. Soc Sci Med 2016;152:119–24. |
| 266 | Habibov N, Auchynnikava A. Quantifying the influence of informal payments on self-rated health: evidence from 26 post-communist countries. Health Policy Plann 2022;37:112–22. |
| 267 | Habibov N, Cheung A. Revisiting informal payments in 29 transitional countries: the scale and socio-economic correlates. Soc Sci Med 2017;178:28–37. |
| 268 | Haker H. The institutional corruption of healthcare bodies. Concilium 2014;5:57–68. |
| 274 | Hjelmeland H, Jaworski K, Knizek B, Marsh I. Problematic advice from suicide prevention experts. Ethical Hum Psychol Psychiatry 2018;20:79–85. |
| 288 | Hope K. Contextualizing corruption in the health sector in developing countries: reflections on policy to manage the risks. World Med Health Policy 2015;7:383–401. |
| 296 | Horodnic A. Trends in informal payments by patients in Europe: a public health policy approach. Front Public Health 2021;9:780337. |
| 297 | Horodnic A, Mazilu S, Oprea L. Drivers behind widespread informal payments in the Romanian public health care system: from tolerance to corruption to socio-economic and spatial patterns. Int J Health Plann Manage 2018;33:e597-e611. |
| 298 | Horodnic A, Williams C, Drugă R, Incaltarau C. Informal payments by patients in Central and Eastern Europe during the COVID-19 pandemic: an institutional perspective. Int J Environ Res Public Health 2021;18:10914. |
| 299 | Houngbo P, et al. The root causes of ineffective and inefficient healthcare technology management in Benin public health sector. Health Policy Techn 2017;6:446–56. |
| 300 | Hsiao A, Vogt V, Quentin W. Effect of corruption on perceived difficulties in healthcare access in sub-Saharan Africa. PLoS ONE 2019;14:1–12. |
| 301 | Hutchinson E, et al. Targeting anti-corruption interventions at the front line: developmental governance in health systems. BMJ Global Health 2020;5:e003092. |
| 311 | Iida K, Proctor R. ‘The industry must be inconspicuous’: Japan Tobacco’s corruption of science and health policy via the Smoking Research Foundation. Tob Control 2018;27:e3-e11. |
| 318 | Incaltarau C, Horodnic A, Williams C, Oprea L. Institutional determinants of informal payments for health services: an exploratory analysis across 117 Countries. Int J Environ Res Public Health 2021;18. |
| 321 | Jesus‐Morales K de, Prasad V. Closed financial loops: when they happen in government, they’re called corruption; in medicine, they’re just a footnote. Hastings Cent Rep 2017;47:9–14. |
| 338 | Jorgensen P. Pharmaceuticals, political money, and public policy: a theoretical and empirical agenda. J Law Med Ethics 2013;41:561–70. |
| 347 | Joudaki H, et al. Improving fraud and abuse detection in general physician claims: a data mining study. Int J Health Policy Manag 2015;5:165–72. |
| 348 | Kagotho N, Bunger A, Wagner K. ‘They make money off of us’: a phenomenological analysis of consumer perceptions of corruption in Kenya’s HIV response system. BMC Health Serv Res 2016;16:468. |
| 357 | Khan N, Puthussery S. Stakeholder perspectives on public-private partnership in health service delivery in Sindh province of Pakistan: a qualitative study. Public Health 2019;170:1–9. |
| 370 | Khoiri A, Hidayat W, Chalidyanto D, Hariadi F. Potential of hospital fraud in the Indonesia national health insurance era. Indian J Med Forensic Med Toxicol 2020;14:1103–7. |
| 373 | Kotoh A, Aryeetey G, van der Geest S. Factors that influence enrolment and retention in Ghana’ national health insurance scheme. Int J Health Policy Manag 2018;7:443–54. |
| 390 | Kumar R, Bhasker S. Administrative corruption in oncology units of developing countries: overview, its impact and possible methods to curb it. J Cancer Policy 2015;5:1–7. |
| 394 | Latysh K, Demidova Y. Corruption offences in medications circulation: investigation problems. Wiad Lek 2021;74:2896–900. |
| 408 | Laurent-Simpson A, Lo C. Risk society online: Zika virus, social media and distrust in the Centers for Disease Control and Prevention. Sociol Health Illn 2019;41:1270–88. |
| 410 | Lê G. Trading legitimacy: everyday corruption and its consequences for medical regulation in southern Vietnam. Med Anthropol Q 2013;27:453–70. |
| 411 | Lexchin J, et al. Combating corruption in the pharmaceutical arena. Indian J Med Ethics 2018;3:234–9. |
| 415 | Li J, et al. Detection of self-reported experiences with corruption on twitter using unsupervised machine learning. Soc Sci Humanit Open 2020;2:100060. |
| 416 | Liang B, Mackey T, Lovett K. Technology and medicine: academic dishonesty and risks to global health. J Commer Biotechnol 2013;19:42–8. |
| 418 | Light D, Lexchin J, Darrow J. Institutional corruption of the pharmaceuticals and the myth of safe and effective drugs. J Law Med Ethics 2013;41:590–600. |
| 420 | Little M, Lipworth W, Kerridge I. An archeology of corruption in medicine. Camb Q Healthc Ethics 2018;27:525–35. |
| 423 | Liu N, Bao G, He A. Does health insurance coverage reduce informal payments? Evidence from the ‘red envelopes’ in China. BMC Health Serv Res 2020;20:1–11. |
| 425 | Mackey T, Vian T, Kohler J. The sustainable development goals as a framework to combat health-sector corruption. Bull World Health Organ 2018;96:634–43. |
| 431 | Maini R, Hotchkiss D, Borghi J. A cross-sectional study of the income sources of primary care health workers in the Democratic Republic of Congo. Hum Resour Health 2017;15:1–15. |
| 435 | Mainuddin R. Bangladesh healthcare corruption and workforce vulnerability amidst the COVID-19 pandemic. Analele Univ din Bucuresti, Ser Stiinte Politice 2021;23:225–43. |
| 436 | Makhinson M, et al. The iatrogenic opioid crisis: An example of ‘institutional corruption of pharmaceuticals’? J Eval Clin Pract 2021;27:1033–43. |
| 437 | Manea T. Medical bribery and the ethics of trust: the Romanian case. J Med Philos 2015;40:26–43. |
| 439 | Marin O, Sen I. Corruption between doctors and pharmacists: Criminal law problems of counteraction. Wiad Lek 2021;74:2901–6. |
| 446 | McKay A. What do campaign contributions buy? Lobbyists’ strategic giving. Int Groups Adv 2018;7:1–18. |
| 453 | Miller J. From bad pharma to good pharma: aligning market forces with good and trustworthy practices through accreditation, certification, and rating. J Law Med Ethics 2013;41:601–10. |
| 466 | Moldovan A, van de Walle S. Gifts or bribes? Public Integrity 2013;15:385–402. |
| 472 | Morgan M, et al. Barriers and facilitators to the provision of optimal obstetric and neonatal emergency care and to the implementation of simulation-enhanced mentorship in primary care facilities in Bihar, India: a qualitative study. BMC Pregnancy Childbirth 2018;18:420. |
| 480 | Mucchielli L. Behind the French controversy over the medical treatment of Covid-19: the role of the drug industry. J Sociol 2020;56:736–44. |
| 483 | Naher N, et al. Absenteeism among doctors in the Bangladesh health system: what are the structural drivers? SSM Qual Res Health 2022;2:100089. |
| 490 | Nara R, Banura A, Foster A. A multi-methods qualitative study of the delivery care experiences of Congolese refugees in Uganda. Matern Child Health J 2020;24:1073–82. |
| 492 | Neerup Handlos L, Fog Olwig K, Bygbjerg I, Norredam M. Return migrants’ experience of access to care in corrupt healthcare systems: the Bosnian example. Int J Environ Res Public Health 2016;13:924. |
| 496 | Nguyen T, et al. Corruption practices in drug prescribing in Vietnam - an analysis based on qualitative interviews. BMC Health Serv Res 2018;18:587. |
| 507 | Noya F, Carr S, Thompson S. Commitments, conditions and corruption: an interpretative phenomenological analysis of physician recruitment and retention experiences in Indonesia. Int J Environ Res Public Health 2022;19:5518. |
| 516 | Ogbozor P, et al. The gendered drivers of absenteeism in the Nigerian health system. Health Policy Plann 2022;37:1267–77. |
| 529 | Osoria A. The COVID-19 pandemic in Puerto Rico: exceptionality, corruption and state-corporate crimes. State Crime J 2021;10:104–25. |
| 538 | Palozzi G, Brunelli S, Falivena C. Higher sustainability and lower opportunistic behaviour in healthcare: a new framework for performing hospital-based health technology assessment. Sustainability 2018;10:3550. |
| 540 | Pandey P, Litoriya R. Implementing healthcare services on a large scale: challenges and remedies based on blockchain technology. Health Policy Techn 2020;9:69–78. |
| 552 | Paul S, Bhatia V. Doctor patient relationship: changing scenario in India. Asian J Med Sci 2016;7:1–5. |
| 555 | Peltier-Rivest D. The prevention and detection of corruption in pharmaceutical companies. Pharm Policy Law 2017;19:17–31. |
| 556 | Peprah P, et al. Why does inaccessibility widely exist in healthcare in Ghana? Understanding the reasons from past to present. J Public Health 2020;28:1–10. |
| 561 | Pieterse P, Lodge T. When free healthcare is not free. Corruption and mistrust in Sierra Leone’s primary healthcare system immediately prior to the Ebola outbreak. Int Health 2015;7:400–4. |
| 565 | Piller C, You J. Hidden conflicts. Science 2018;361:16–20. |
| 582 | Purohit B, et al. Opening the black box of transfer systems in public sector health services in a Western state in India. BMC Health Serv Res 2016;16:419. |
| 587 | Radević I, Alfirević N, Lojpur A. Corruption, public trust and medical autonomy in the public health sector of Montenegro: taking stock of the COVID-19 influence. PLoS ONE 2022;17:e0274318. |
| 590 | Rahmani Z, Brekke M. Antenatal and obstetric care in Afghanistan - a qualitative study among health care receivers and health care providers. BMC Health Serv Res 2013;13:1–9. |
| 592 | Rajaković D, Milovanović A. Corruption as a jeopardising factor of health care system development in the Republic of Serbia. Megatrend Rev 2019;16:111–21. |
| 594 | Rattani S, Dahlke S, Cameron B. Cancer care in Pakistan: a descriptive case study. Glob Qual Nurs Res 2022;9:23333936221080988. |
| 595 | Raventós P, Zolezzi S. Electronic tendering of pharmaceuticals and medical devices in Chile. J Bus Res 2015;68:2569–78. |
| 596 | Rawlinson P. Immunity and impunity: corruption in the state-pharma nexus. Int J Crime Justice Soc Democr 2017;6:86–99. |
| 603 | Reznik O, Bondarenko O, Utkina M, Horobets N. Corruptive incomings laundering in the medical sphere. Wiad Lek 2021;74:1250–5. |
| 608 | Rispel L, Jager P de, Fonn S. Exploring corruption in the South African health sector. Health Policy Plann 2016;31:239–49. |
| 611 | Rodwin M. Five un-easy pieces of pharmaceutical policy reform. J Law Med Ethics 2013a;41:581–9. |
| 613 | Rodwin M. Rooting out institutional corruption to manage inappropriate off-label drug use. Edmond J Safra Center for Ethics Working Paper Series 2013b. |
| 617 | Rose-Ackerman S. Corruption and COVID-19. Eunomia 2021;20:16–36. |
| 618 | Rose-Ackerman S, Yingqi T. Corruption in the procurement of pharmaceuticals and medical equipment in China: the incentives facing multinationals, domestic firms and hospital officials. UCLA Pac Basin Law J 2014;32:1–53. |
| 631 | Schaaf M, Dasgupta J. ‘Our fear is finished,’ but nothing changes: efforts of marginalized women to foment state accountability for maternal health care in a context of low state capacity. BMC Public Health 2019;19:732. |
| 635 | Schipani C, Junhai Liu, Haiyan X. Doing business in a connected society: the GSK bribery scandal in China. Univ Ill Law Rev 2016;2016:63–102. |
| 639 | Sekalala S, Masud H, Bosco R. Human rights mechanisms for anti-corruption, transparency and accountability: enabling the right to health. Glob Health Action 2020;13:1–24. |
| 640 | Serneels P, Lievens T. Microeconomic institutions and personnel economics for health care delivery: a formal exploration of what matters to health workers in Rwanda. Hum Resour Health 2018;16:7. |
| 644 | Shi J, et al. Moving towards a better path? A mixed-method examination of China’s reforms to remedy medical corruption from pharmaceutical firms. BMJ Open 2018;8:e018513. |
| 654 | Sismondo S. Key opinion leaders and the corruption of medical knowledge: what the Sunshine Act will and won’t cast light on. J Law Med Ethics 2013;41:635–43. |
| 656 | Snyder J, et al. Outbound medical tourism from Mongolia: a qualitative examination of proposed domestic health system and policy responses to this trend. BMC Health Serv Res 2015;15:187. |
| 662 | Sommersguter-Reichmann M, Stepan A. Hospital physician payment mechanisms in Austria: do they provide gateways to institutional corruption? Health Econ Rev 2017;7:11. |
| 664 | Somogyvári M. The costs of organisational injustice in the Hungarian health care system. J Bus Ethics 2013;118:543–60. |
| 670 | Sripad P, et al. Determining a trusting environment for maternity care: a framework based on perspectives of women, communities, service providers, and managers in peri-urban Kenya. Front Glob Womens Health 2022;3:818062. |
| 673 | Stepurko T, et al. Informal payments for health care services: the case of Lithuania, Poland and Ukraine. J Eurasian Stud 2015;6:46–58. |
| 674 | Stepurko T, et al. Patterns of informal patient payments in Bulgaria, Hungary and Ukraine: a comparison across countries, years and type of services. Health Policy Plann 2017;32:453–66. |
| 692 | Tapia M, Cabrita M, Flores D, Tapia C. Neutrosociology for analysing public procurement in Ecuador around the health emergency. Neutrosophic Sets Syst 2021;44:333–41. |
| 696 | Teremetskyi V, et al. Corruption and strengthening anti-corruption efforts in healthcare during the pandemic of Covid-19. Med Leg J 2021;89:25–8. |
| 700 | Tillmanns C. Compliance: 18 months of the German act on fighting corruption in the healthcare dector. Eur Pharm Law Rev 2018;2:26–31. |
| 710 | Tuchilus G, Stanculescu D, Roman M, Huru D. The gift game in healthcare sector – the case of Romania. Econ Comput Econ Cybern Stud Res 2022;56:5–20. |
| 711 | Tumlinson K, et al. Informal payments for family planning: prevalence and perspectives of women, providers, and health sector key informants in western Kenya. Sex Reprod Health Matters 2021;29:1–17. |
| 714 | Uddin J, Momtaz S, Islam M. State obligation towards the fulfillment of the right to health: a study in Bangladesh perspective. Mediterr J Soc Sci 2013;4:73. |
| 717 | Valverde J. The pharmaceuticals industry in trouble. Pharm Policy Law 2013;15:51–69. |
| 721 | Vega R, Maya A. Operating at the edge of il/legality: systemic corruption in Mexican health care. J Latin Amer Carib Anth 2021;26:46–64. |
| 723 | Vian T, Mcintosh N, Grabowski A. ‘It keeps us from putting drugs in pockets’: how a public-private partnership for hospital management may help curb corruption. Perm J 2017;21:112. |
| 729 | Wafula F, Molyneux C, Mackintosh M, Goodman C. Protecting the public or setting the bar too high? Understanding the causes and consequences of regulatory actions of front-line regulators and specialised drug shop operators in Kenya. Soc Sci Med 2013;97:220–7. |
| 740 | Widmer P, Zurlinden N. Ministers engage in favoritism too. J Publ Econ 2022;213:104707. |
| 743 | Williams C, Horodnic I, Horodnic A. Who is making informal payments for public healthcare in East-Central Europe? An evaluation of socio-economic and spatial variations. East J Eur Stud 2016;7:49–61. |
| 758 | Zamboni Y, Litschig S. Audit risk and rent extraction: evidence from a randomized evaluation in Brazil. J Dev Econ 2018;134:133–49. |
| 762 | Zhu W, Wang L, Yang C. Corruption or professional dignity: an ethical examination of the phenomenon of ‘red envelopes’ (monetary gifts) in medical practice in China. Dev World Bioeth 2018;18:37–44. |
